# Substance Use Disorder as a Risk Factor for Postpartum Depression: A Retrospective Chart Review in a Community Hospital

**DOI:** 10.7759/cureus.68450

**Published:** 2024-09-02

**Authors:** Eduardo D Espiridion, Diane Lee

**Affiliations:** 1 Psychiatry, Drexel University College of Medicine, Philadelphia, USA; 2 Psychiatry, Reading Hospital - Tower Health, West Reading, USA

**Keywords:** depression after birth, sud, substance use disorder, ppd, postpartum depression

## Abstract

Objective: This retrospective study examines the relationship between postpartum depression (PPD) and substance use disorder (SUD) in a community hospital setting.

Methods: This retrospective chart review explored the association between SUD and PPD in a community hospital. Data from January 2016 to December 2018 were extracted from electronic medical records (EPIC EMR (Epic Systems Corporation, Verona, WI)), identifying mothers with PPD (n = 99) using billing code F53.0. Substance use disorder was assessed using diagnostic codes F10-F19. Odds ratios (OR), relative risk (RR), and chi-square tests were calculated to quantify and assess the significance of the association between SUD and PPD. Ethical approval was obtained from the Institutional Review Board (IRB).

Results: Among 2,517 deliveries during the study period, 51 cases of PPD co-occurred with SUD. Mothers with SUD had a 4.3 times higher risk of PPD compared to those without SUD (OR = 4.8), highlighting a significant association.

Discussion: These findings emphasize the importance of screening for PPD and SUD in pregnant and postpartum women, especially in community healthcare settings where routine screening may be limited. Targeted interventions can mitigate adverse effects on maternal and infant well-being.

## Introduction

Postpartum depression (PPD) is a condition characterized by profound feelings of despair or anxiety that significantly impede women’s daily functioning. Occurring anywhere from one day to one year after childbirth, PPD typically manifests most commonly within one to three weeks postpartum, affecting up to one in seven women. This condition has a wide range of adverse effects on both mothers and their newborns, including disruptions in feeding and sleep patterns. Additionally, affected mothers are less likely to engage in enriching activities with their infants, such as reading, singing, and storytelling [1], and may even struggle to respond to their babies' positive cues like smiles. Disturbingly, infants can begin to mirror the depressed behavior of their mothers [2]. Furthermore, individuals who have experienced PPD face a significantly higher risk, twice as likely, of developing depression even four years after childbirth and are more prone to chronic illnesses, such as heart disease, diabetes, and hypertension [3].

Although the precise causes of PPD are multifaceted and not fully elucidated, prior research has identified numerous risk factors that increase a woman's vulnerability. Notably, a history of depression escalates the risk of PPD by 20-fold compared to women with no such history [4]. While depression history is the most prominent predictor among psychiatric disorders, anxiety also significantly heightens the risk of PPD one year postpartum. Additionally, panic disorders, obsessive-compulsive disorders, post-traumatic stress disorders, and eating disorders demonstrate positive associations with PPD [5, 6].

Another psychiatric condition linked to PPD is substance use disorder (SUD). The Diagnostic and Statistical Manual (DSM-V) criteria for SUD encompass a range of symptoms resulting from continued substance use despite adverse consequences, including impaired control, physical dependence, social problems, and risky behavior [7]. Substances involved can range from alcohol, opioids, cocaine, cannabis, amphetamines, barbiturates, and hallucinogens. Diagnosis of SUD requires a comprehensive evaluation involving self-reports, collateral information from family and friends, physical examinations by physicians, and laboratory tests [8]. Notably, the percentage of adults aged between 18 and 49 diagnosed with both mental illness and SUD has surged over the past decade [9].

Substance use disorder was identified as a risk factor for treatment-resistant depression in patients with major depressive disorder [10]. Lifetime drug use is also correlated with postpartum stress and anxiety [11], with those who experienced adverse childhood experiences being notably susceptible to any postpartum psychiatric disorder [12]. Previous studies have underscored the association between substance use disorder and PPD, emphasizing the critical role of social networks and support [13, 14]. Other studies have highlighted risk factors like single marital status, tobacco or illegal drug use during pregnancy, and a history of substance abuse in relation to PPD [15]. Despite estimates indicating that 8%-11% of pregnant women aged 15 to 44 use illicit drugs, tobacco, or alcohol monthly, the true extent of the link between SUD and PPD remains challenging to ascertain due to the potential underreporting of maternal substance abuse [16]. Consequently, this study aims to assess the magnitude of the association between PPD and SUD within a community hospital setting.

## Materials and methods

Study design

This retrospective study aims to investigate SUD as a risk factor for PPD. Data was collected through a chart review of patients at a community hospital in Pennsylvania.

Data collection

Data collection took place between January 1, 2016, and December 31, 2018, excluding the COVID-19 pandemic period. The study population included mothers diagnosed with PPD during this timeframe. The ambulatory Clinical Standards Program Manager of Maternal Child Health and Specialty facilitated data collection.

Charts of all mothers diagnosed with PPD (identified by billing code F53.0) during the specified period were retrieved from the hospital's electronic medical records (EPIC EMR (Epic Systems Corporation, Verona, WI)). The total number of identified patients diagnosed with PPD during this period was 99. There were no specific exclusion criteria. In handling missing data for this study, initially, the dataset was screened for completeness. Each variable of interest, including PPD diagnosis, SUD diagnosis, and demographic information, was examined for missing entries. Records with missing data on key variables (i.e., PPD or SUD diagnosis) were excluded from the final analysis. This ensured that the analysis was conducted only on complete cases, minimizing potential biases introduced by incomplete data.

Screening for substance use disorder

Patients with PPD were further screened for co-occurring SUD using relevant diagnostic codes (F10-F19). The substances considered included opioids, alcohol, nicotine, cannabis, and others (unspecified). 

Demographic and clinical variables

The extracted data included demographic information (age, postal zip code, ethnicity) and clinical details (date of PPD diagnosis, presence of SUD). The use of medical record numbers (MRNs) ensured data integrity while maintaining patient confidentiality.

Statistical analysis

The study employed several statistical methods to assess the relationship between SUD and PPD. First, the prevalence of PPD was calculated for mothers with and without SUD. An odds ratio (OR) was then computed to quantify the strength of the association between SUD and PPD, with a confidence interval (CI) of 95% to estimate the precision of the OR. Additionally, the relative risk (RR) was calculated to compare the risk of developing PPD in mothers with SUD to those without.

To determine the statistical significance of the observed association, a chi-square test of independence was conducted, comparing the observed frequencies of PPD in mothers with and without SUD. The test generated a chi-square statistic and a corresponding p-value, with a significance level set at 0.05.

Ethical considerations

This study was approved by the Institutional Review Board (IRB). To protect patient privacy, no identifiable information was used in the analysis. All data were securely stored and processed on hospital computers with encryption.

## Results

We have retrospectively reviewed the medical records of patients who were admitted to the obstetrics unit of this community hospital between 2016 and 2019. There were a total of 99 patients with a diagnosis of PPD between the three years (Table 1). 

**Table 1:** Number of postpartum depression (PPD) diagnoses per year The number of PPD diagnoses per year stayed consistent over time.

Year	PPD diagnoses
2016-2017	34
2017-2018	34
2018-2019	31

**Figure 1:**
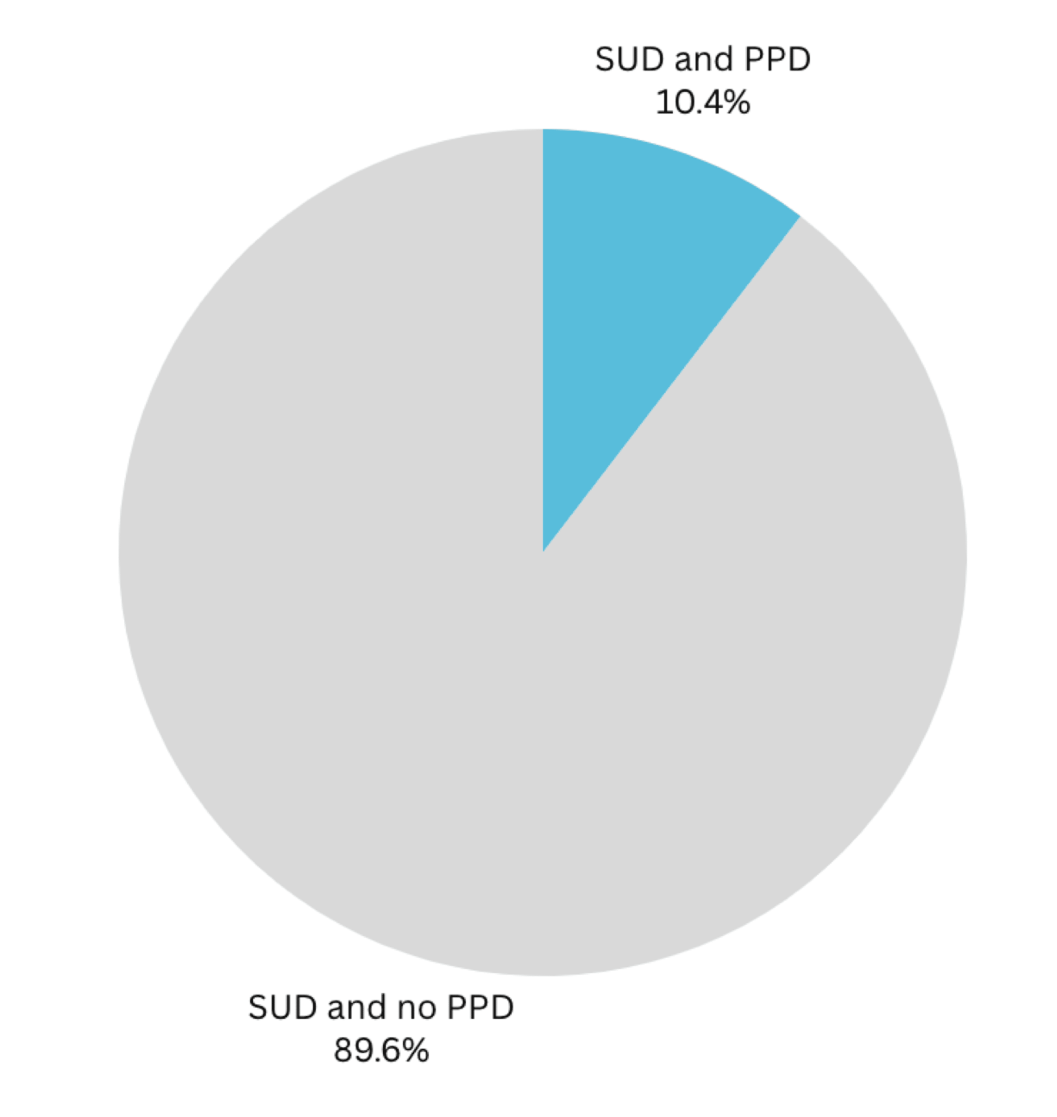
Postpartum depression (PPD) in substance use disorder (SUD) patients There were 489 mothers with a SUD diagnosis; 51 were also diagnosed with PPD during or shortly after their pregnancy. Prevalence rate was 10.4%.

We were also able to identify 489 postpartum patients with a SUD diagnosis during the study period. Out of these patients, 51 were also diagnosed with PPD during or shortly after their pregnancy (Figure 1). The diagnoses of both PPD and SUD were made by attending physicians and consulting psychiatrists.

There were a total of 2,517 patients who were admitted to the obstetrics floor during the study period; PPD was present in 99 patients, while in 2,418 patients, PPD was absent (Table 2). 

**Table 2:** Postpartum depression (PPD) and substance use disorder (SUD) diagnoses This table shows the total number of PPD and non-PPD patients, with and without SUD, who were admitted during the study period.

	PPD present	PPD absent
SUD	51	438
No SUD	48	1980
Total	99	2418

Statistical analysis of our data revealed significant findings that underscore the association between SUD and PPD. Among the 2,517 deliveries recorded during the study period, the overall prevalence of PPD was 3.93%. The OR was 4.8 and the RR was 4.3. However, when stratified by SUD status, the prevalence of PPD was markedly higher in mothers with SUD at 10.43%, compared to just 2.37% in those without SUD. This disparity was further quantified through the calculation of the OR, which demonstrated that mothers with SUD were approximately 4.8 times more likely to develop PPD than their counterparts without SUD. The RR further supported this association, showing that the risk of developing PPD was 4.4 times higher for mothers with SUD.

To evaluate the statistical significance of this association, a chi-square test was conducted. The results indicated a chi-square value of 65.67 with an exceedingly low p-value of 5.34×10^−16^, strongly suggesting that the observed association between SUD and PPD is not due to chance. These findings emphasize the need for enhanced screening and early intervention strategies targeting SUD in pregnant and postpartum women to mitigate the elevated risk of PPD and improve maternal and infant health outcomes.

## Discussion

This study aimed to explore the relationship between SUD and PPD within a community hospital setting. The data revealed a significant association between these two conditions, with mothers experiencing SUD exhibiting a markedly higher risk of developing PPD. Specifically, the risk of PPD among mothers with SUD was 10.4%, and the RR was calculated to be 4.3, indicating that these individuals are 4.3 times more likely to develop PPD than those without SUD. The OR of 4.8 further underscores the robustness of this association. This also mirrors other studies, which found an OR of 3.67 between these two variables [17], reinforcing the robustness and consistency of the observed association between SUD and PPD risk. This consistency across studies highlights the need for a comprehensive approach to maternal mental health that integrates substance use screening and intervention strategies.

Implications of the findings

The findings from this study emphasize the critical need for comprehensive screening and intervention strategies for PPD and SUD, particularly in community healthcare settings where routine screenings may be limited. Given the significant association between SUD and PPD, integrating mental health assessments into prenatal and postpartum care is essential. Early identification and intervention for women with SUD who are pregnant or planning to conceive can facilitate timely and effective treatments, potentially mitigating the adverse impacts on both maternal and infant well-being [18].

Limitations

One notable limitation of this study is the potential underreporting of PPD cases due to the lack of routine screening prior to 2019. This highlights a broader issue in community healthcare settings, where systematic screening for PPD and SUD may not be consistently implemented. Addressing this gap requires systemic changes to healthcare protocols, ensuring that all pregnant and postpartum women undergo comprehensive screenings for these conditions. In addition, our study did not look at other mental health disorders along with SUD that could have affected PPD onset as well. 

Recommendations for healthcare providers

Healthcare providers should prioritize the integration of mental health screenings into routine obstetric care. Implementing evidence-based screening protocols for PPD and SUD, especially among vulnerable populations, is crucial. By incorporating these screenings into standard prenatal and postpartum care, healthcare systems can ensure early identification and intervention, which are vital for improving maternal and infant health outcomes. Early detection and appropriate interventions, such as psychotherapy or pharmacological treatments, have been shown to significantly improve health outcomes for both mother and child [19, 20].

Future research directions

The study provides valuable demographic data, including age, postal zip code, and ethnicity, which are crucial for understanding the broader context of PPD and SUD. Socioeconomic factors, such as access to healthcare, social support networks, and socioeconomic status, significantly influence the prevalence and management of these conditions. Future research should delve deeper into how these factors affect the risk and management of PPD and SUD, providing a more holistic understanding of the challenges faced by affected women.

Understanding the underlying mechanisms that contribute to the heightened risk of PPD among individuals with SUD is essential for developing effective interventions. The comorbidity of SUD and PPD may be influenced by a range of biological, psychological, and social factors. For instance, substance use can exacerbate existing mental health conditions or create new psychological stressors, leading to a higher likelihood of developing PPD. Additionally, the social stigma and isolation often associated with substance use can further compound mental health challenges [21]. Future research should explore these mechanisms in greater detail to inform the development of tailored interventions.

Further research is warranted to explore the complex relationship between SUD and PPD. Longitudinal studies could provide more detailed insights into how these conditions develop and interact over time. Additionally, efforts to develop and test tailored interventions for women with dual diagnoses (SUD and PPD) are needed. Such initiatives can inform evidence-based practices aimed at reducing the burden of maternal mental health disorders within vulnerable populations. Exploring the role of social support networks, access to healthcare, and other socioeconomic factors will be crucial in developing comprehensive strategies to address these co-occurring disorders.

## Conclusions

In conclusion, our retrospective study highlights a significant association between substance use disorder and postpartum depression in a community hospital setting, with mothers experiencing SUD being 4.3 times more likely to develop PPD. These findings underscore the importance of integrating mental health screenings for PPD and SUD into routine prenatal and postpartum care, especially in community healthcare settings. Early identification and timely interventions can mitigate the adverse effects on maternal and infant health. Further research is needed to explore the mechanisms linking SUD and PPD and to develop targeted interventions for women with dual diagnoses. Advancing our understanding of these co-occurring disorders will enhance maternal mental health outcomes and overall well-being.

## References

[1] Paulson JF, Dauber S, Leiferman JA (2006). Individual and combined effects of postpartum depression in mothers and fathers on parenting behavior. Pediatrics.

[2] Beck CT (1995). The effects of postpartum depression on maternal-infant interaction: a meta-analysis. Nurs Res.

[3] Abdollahi F, Zarghami M (2018). Effect of postpartum depression on women's mental and physical health four years after childbirth. East Mediterr Health J.

[4] Silverman ME, Reichenberg A, Savitz DA (2017). The risk factors for postpartum depression: a population-based study. Depress Anxiety.

[5] Nakano M, Sourander A, Luntamo T, Chudal R, Skokauskas N, Kaneko H (2020). Early risk factors for postpartum depression: a longitudinal Japanese population-based study. J Affect Disord.

[6] Johansen SL, Stenhaug BA, Robakis TK, Williams KE, Cullen MR (2020). Past psychiatric conditions as risk factors for postpartum depression: a nationwide cohort study. J Clin Psychiatry.

[7] American Psychiatric Association (2013). The Diagnostic and Statistical Manual of Mental Disorders, Fifth Edition.

[8] Ruiz P, Strain EC, Langrod JG (2014). The Substance Abuse Handbook.

[9] (2019). Key substance use and mental health indicators in the United States: results from the 2019 National Survey on Drug Use and Health. https://www.samhsa.gov/data/sites/default/files/reports/rpt29393/2019NSDUHFFRPDFWHTML/2019NSDUHFFR090120.htm.

[10] Brenner P, Brandt L, Li G, DiBernardo A, Bodén R, Reutfors J (2020). Substance use disorders and risk for treatment resistant depression: a population-based, nested case-control study. Addiction.

[11] Prevatt BS, Desmarais SL, Janssen PA (2017). Lifetime substance use as a predictor of postpartum mental health. Arch Womens Ment Health.

[12] Meltzer-Brody S, Larsen JT, Petersen L (2018). Adverse life events increase risk for postpartum psychiatric episodes: a population-based epidemiologic study. Depress Anxiety.

[13] Pajulo M, Savonlahti E, Sourander A, Helenius H, Piha J (2001). Antenatal depression, substance dependency and social support. J Affect Disord.

[14] Kuo C, Schonbrun YC, Zlotnick C, Bates N, Todorova R, Kao JC, Johnson J (2013). A qualitative study of treatment needs among pregnant and postpartum women with substance use and depression. Subst Use Misuse.

[15] Bryan TL, Georgiopoulos AM, Harms RW, Huxsahl JE, Larson DR, Yawn BP (1999). Incidence of postpartum depression in Olmsted County, Minnesota. A population-based, retrospective study. J Reprod Med.

[16] Dennis CL, Vigod S (2013). The relationship between postpartum depression, domestic violence, childhood violence, and substance use: epidemiologic study of a large community sample. Violence Against Women.

[17] Pacho M, Aymerich C, Pedruzo B (2023). Substance use during pregnancy and risk of postpartum depression: a systematic review and meta-analysis. Front Psychiatry.

[18] Goler NC, Armstrong MA, Osejo VM, Hung YY, Haimowitz M, Caughey AB (2012). Early start: a cost-beneficial perinatal substance abuse program. Obstet Gynecol.

[19] Terrone G, Bianciardi E, Fontana A (2023). Psychological characteristics of women with perinatal depression who require psychiatric support during pregnancy or postpartum: a cross-sectional study. Int J Environ Res Public Health.

[20] Saharoy R, Potdukhe A, Wanjari M, Taksande AB (2023). Postpartum depression and maternal care: exploring the complex effects on mothers and infants. Cureus.

[21] Ross LE, Dennis CL (2009). The prevalence of postpartum depression among women with substance use, an abuse history, or chronic illness: a systematic review. J Womens Health (Larchmt).

